# The impact of inflammatory burden index on the prognosis in acute decompensated heart failure: evidence from a cohort study in Jiangxi, China

**DOI:** 10.3389/fcvm.2025.1604094

**Published:** 2025-10-10

**Authors:** Kun Jiang, Guoan Jian, Zihao Lu, Shiming He, Xinfang Huang, Lin Xie, Shuhua Zhang, Qun Wang, Hengcheng Lu, Zhiyu Xiong, Zhiting Wu, Guotai Sheng, Yang Zou, Aimin Xie, Hengli Lai, Wei Wang

**Affiliations:** ^1^ Jiangxi Medical College, Nanchang University, Nanchang, China; ^2^ Jiangxi Cardiovascular Research Institute, Jiangxi Provincial People's Hospital, The First Affiliated Hospital of Nanchang Medical College, Nanchang, China; ^3^ Department of Cardiology, Jiangxi Provincial People’s Hospital, The First Affiliated Hospital of Nanchang Medical College, Nanchang, China; ^4^ Department of Cardiovascular Surgery, Jiangxi Provincial People’s Hospital, The First Affiliated Hospital of Nanchang Medical College, Nanchang, China

**Keywords:** inflammatory burden index, acute decompensated heart failure, Chinese, prognosis, IBI

## Abstract

**Objective:**

Acute decompensated heart failure (ADHF) is the most common and severe type of HF. The aim of this study is to evaluate the impact and predictive value of a novel inflammatory marker, the inflammatory burden index (IBI), on the 30-day mortality and adverse prognosis in patients with ADHF.

**Methods:**

This retrospective cohort study included 1,241 ADHF patients from Jiangxi Provincial People's Hospital between 2018 and 2024. The IBI was calculated as C-reactive protein × (neutrophil count/lymphocyte count). In the event analysis, the study outcome was defined as the 30-day mortality rate after hospital admission in ADHF patients. Multivariable Cox regression and receiver operating characteristic curve analysis were used to assess the impact and predictive value of the IBI on 30-day mortality. Additionally, subgroup analyses were performed to determine the risk dependency of the IBI within specific populations.

**Results:**

During the 30-day observation period, a total of 108 death events (8.70%) were recorded. When the study population was stratified into tertiles based on the IBI, the 30-day mortality rates were 1.93%, 4.60%, and 19.57%, respectively. Multivariable Cox regression analysis revealed a significant positive association between the IBI and 30-day mortality in ADHF patients (HR per SD increase: 1.29, 95% CI: 1.15–1.46). Compared to ADHF patients with a low IBI (T1), those with a high IBI (T3) showed a 368% higher risk of 30-day mortality (HR: 4.68, 95% CI: 1.06–13.73). Subgroup analysis revealed a significant interaction between the IBI and 30-day mortality in ADHF patients across sex subgroups (*P*-interaction < 0.05). In particular, compared to male patients, female ADHF patients exhibited a significantly higher risk of IBI-related in-hospital all-cause mortality (HR: 1.52 vs. 1.33). Receiver operating characteristic analysis further demonstrated that the novel inflammatory marker IBI had the highest AUC value (0.80) compared to conventional inflammatory markers, including C-reactive protein, white blood cell count, neutrophil count, lymphocyte count, and monocyte count.

**Conclusion:**

The cohort study conducted in Jiangxi, China, revealed that the novel inflammatory marker IBI is significantly positively associated with 30-day mortality in ADHF patients and demonstrated strong predictive value. Incorporating IBI into the clinical management of ADHF patients may hold significant potential for preventing further disease deterioration.

## Graphical Abstract

**Figure:**
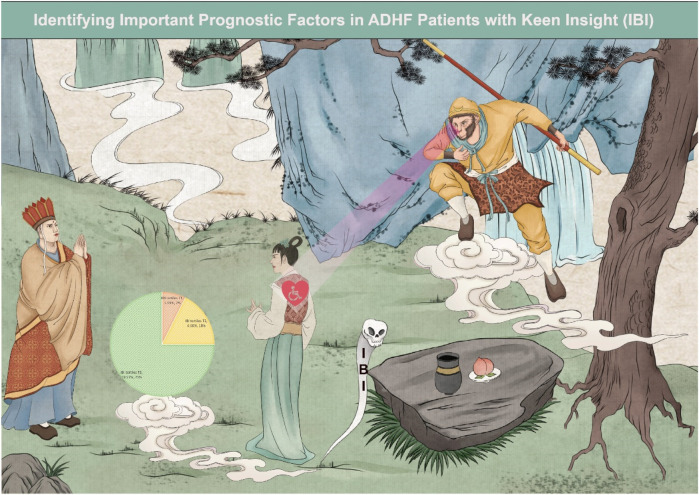
Explanation: In this graphic abstract, we drew inspiration from characters in Journey to the West, a classic Chinese literary masterpiece. Here, the “White Bone Demon” (Bai Gu Jing; a shape-shifting demoness) symbolizes ADHF patients, while 'Sun Wukong' (the Monkey King) represents physicians. As depicted, the physician, akin to Sun Wukong, employs his fiery golden eyes (a metaphor for clinical acumen) to identify the IBI (represented by a skull icon) as the key risk factor in the ADHF patient.

## Introduction

With the intensification of global aging, heart failure (HF) is becoming an increasingly serious public health issue. According to the Global Burden of Disease data report, the number of HF patients worldwide has exceeded 55 million as of 2021 (1). Among the various types of HF, Acute decompensated HF (ADHF) represents the most prevalent and severe form, characterized by new or worsening clinical symptoms and signs of HF (2–4). ADHF is not only one of the most frequent causes of hospitalization among the elderly population but is also associated with a significantly elevated risk of short-term adverse clinical outcomes. Studies have demonstrated that ADHF has an in-hospital mortality rate of approximately 5.3%–7.5% and a one-year mortality rate of around 25%, imposing a significant disease burden on both patients and society (4–8). Despite recent key advancements in the treatment of HF, the management of ADHF patients remains one of the greatest challenges for cardiologists (9, 10). Therefore, it is crucial to identify clinically useful biomarkers that can predict the prognosis of ADHF at an early stage, thereby optimizing clinical decision-making.

Inflammation plays a critical role in HF progression through multiple mechanisms. Compared to chronic HF, inflammatory activation is more pronounced in acute HF patients, and inflammatory levels are significantly associated with adverse outcomes (11–16). Therefore, early assessment of inflammation holds significant importance for ADHF patients. In recent years, a novel inflammatory indicator known as the inflammatory burden index (IBI), calculated based on C-reactive protein (CRP), neutrophil count, and lymphocyte count, has garnered the attention of numerous researchers. They have discovered that IBI may possess high application potential as an inflammatory indicator and holds significant value in the prognostic assessment of various chronic and oncological diseases (17–32). For chronic diseases, existing research evidence indicates that the IBI is applicable to the prognostic assessment of osteoarthritis, rheumatoid arthritis, inflammatory airway diseases, ischemic stroke, and intracerebral hemorrhage (28–32). IBI has also been identified as an independent risk factor for cardiovascular diseases and can be utilized for risk assessment in HF, angina pectoris, coronary heart disease (CHD), and stroke (33). Given that the progression of ADHF is significantly associated with the activation of inflammation (11–16), further elucidating the relationship between the IBI and ADHF prognosis may provide valuable insights for disease management. To address this issue, this study aims to evaluate the impact and predictive value of IBI on 30-day mortality prognosis in ADHF patients using the ADHF cohort from Jiangxi, China.

## Methods

### Study population and design

The data used in this survey comes from Jiangxi-acute decompensated heart failure study II. This is a cohort study initiated by Jiangxi Provincial People's Hospital, consecutively enrolling 3,484 patients with ADHF admitted to the Jiangxi Provincial People's Hospital from January 2018 to January 2024. The primary objective of this project is to establish a high-quality cohort of ADHF patients, effectively utilize their clinical record data during hospitalization, and explore new methods for early risk stratification to improve the adverse prognosis of ADHF patients. In this study, the diagnosis of ADHF was based on the ESC and ACC/AHA/HFSA Heart Failure Guidelines (2, 3), incorporating clinical symptoms, physical signs, and laboratory findings. The diagnostic criteria were as follows: The presence of at least one sign of HF: (a) Elevated N-terminal pro-brain natriuretic peptide (NT-proBNP); (b) Pulmonary edema detected by physical examination or chest x-ray; (c) Abnormal cardiac structure and/or function as indicated by echocardiography. The presence of at least one symptom of worsening HF: (a) Systemic venous congestion; (b) Dyspnea; (c) Insufficient tissue perfusion.

**Figure 1:**
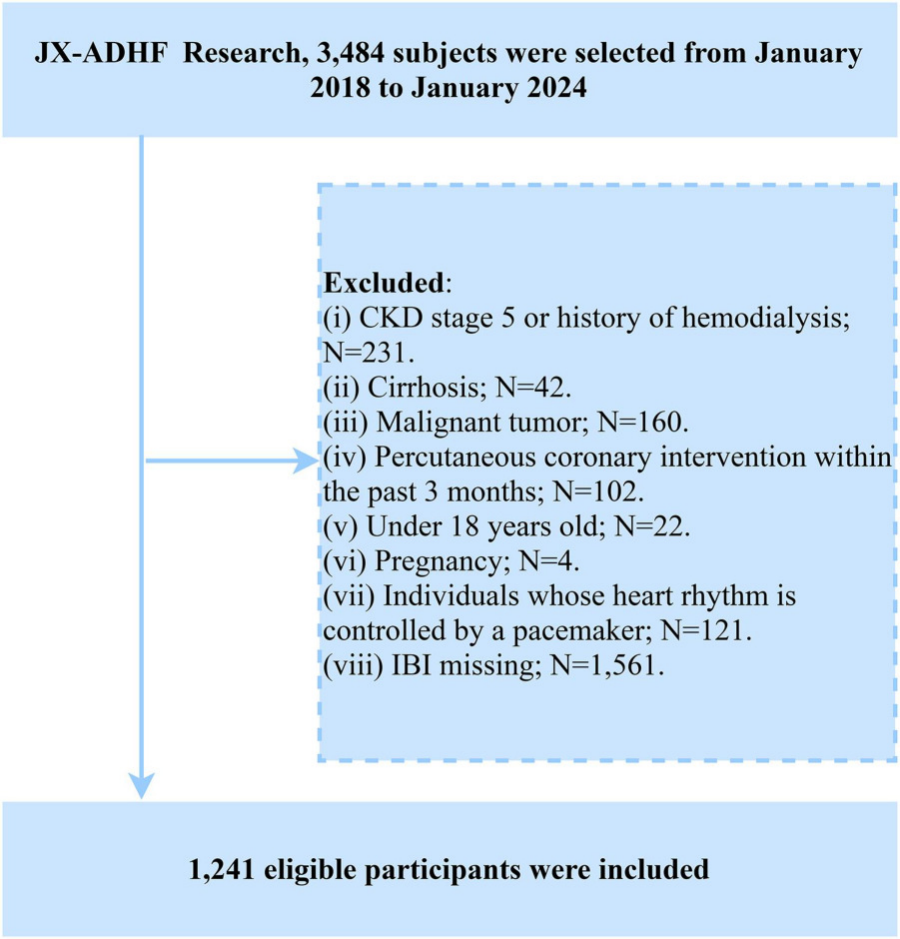
Flow chart for inclusion and exclusion of study participants. ADHF, acute decompensated heart failure; IBI, inflammatory burden index; CKD, chronic kidney disease.

In the current study, we established the following exclusion criteria based on the research objectives: (i) To account for the potential impact of additional fluid and sodium retention, we excluded patients with uremia or a history of hemodialysis (*n* = 231) and those with liver cirrhosis (*n* = 42); (ii) Considering the potential influence on life expectancy, we excluded patients with malignant tumors (*n* = 160); (iii) Due to the significant role of reperfusion therapy in short-term prognosis, participants who had undergone percutaneous coronary intervention (PCI) within the past 3 months were excluded (*n* = 102); (iv) Participants under the age of 18 (*n* = 22); (v) Pregnant individuals (*n* = 4); (vi) Individuals with pacemaker-controlled heart rhythms, as their heart rates were not expected to be regulated by autonomic nervous control (*n* = 121). Additionally, we excluded participants with missing IBI data (*n* = 1,561). Ultimately, 1,241 patients with ADHF were included in the analysis. Given the high rate of missing IBI data in this study, we conducted a systematic evaluation of baseline characteristic differences between the complete-case group and the missing-data group prior to formal analysis. As shown in Supplementary Table S1, no statistically significant differences were observed between the two groups across most baseline characteristics (*P* > 0.05), suggesting that the missing data mechanism aligns with the missing at-random assumption. This finding provides methodological assurance regarding data quality for subsequent analyses. A detailed flowchart of the study population screening process is shown in Figure 1.

### Ethical approval

This study adhered to the ethical principles outlined in the Declaration of Helsinki. The use of research data strictly complied with ethical review requirements, and authorization was obtained from patients and their families. The study protocol was approved by the Ethics Committee of Jiangxi Provincial People's Hospital (IRB: 2024-01). The study followed the Strengthening the Reporting of Observational Studies in Epidemiology reporting guidelines to ensure transparency and scientific rigor of the findings (34).

### Data collection

The baseline data for this study were collected by two trained researchers from the hospital's electronic medical record system, with cross-verification to ensure accuracy. The specific details are as follows: (i) Demographic and clinical data: sex, age, drinking status, smoking status, comorbidities [including hypertension, diabetes, stroke, and CHD], cardiac function (New York Heart Association classification: NYHA), blood pressure data [measured using an Omron automatic blood pressure monitor (HBP-1300) in a quiet environment or at the bedside] and medication information during hospitalization [Includes the use of beta-blockers, diuretics, angiotensin-converting enzyme inhibitors (ACEI)/angiotensin receptor inhibitors (ARB)/angiotensin receptor neprilysin inhibitors (ARNI), and vasopressor medications]. (ii) Echocardiographic examination: Left ventricular ejection fraction (LVEF). (iii) Laboratory test data: The biochemical indicators measured included albumin, alanine aminotransferase, aspartate aminotransferase (AST), creatinine (Cr), uric acid (UA), total cholesterol (TC), triglycerides (TG), low-density lipoprotein cholesterol (LDL-C), high-density lipoprotein cholesterol (HDL-C), and fasting plasma glucose (FPG). Additionally, other assessed parameters included white blood cell (WBC) count, red blood cell (RBC) count, platelet count, CRP, neutrophil count, lymphocyte count, and NT-proBNP. All blood samples were collected within 24 h of hospital admission, adhering strictly to the timing requirements for laboratory results. For liver enzymes, lipid profiles, and FPG, venous blood samples were collected either at admission under fasting conditions or on the morning of the second day after admission.

### IBI calculation

IBI = CRP × (neutrophil count/lymphocyte count) (17).

### Study outcomes

The primary endpoint of this study was all-cause mortality within 30 days after hospital admission in patients with ADHF. The 30-day survival status of all participants was tracked by trained medical staff through multiple methods, including text messages, phone calls, and face-to-face follow-ups during outpatient clinics or hospital admissions.

### Statistical analysis

All statistical analyses in this study were performed using R software (version 4.2.1) and Empower® software (version 2.0). Statistical significance was defined as a two-sided *p*-value < 0.05.

First, we stratified ADHF patients into tertiles (low, moderate, and high) based on IBI, which were determined by calculating the 33.33% and 66.67% percentiles of IBI values. Baseline variables were described according to their type and distribution: categorical variables were expressed as counts (%), while continuous variables were expressed as mean ± standard deviation (SD) or median (interquartile range), as appropriate. Group differences were analyzed using chi-square tests, one-way ANOVA, or non-parametric tests, as appropriate.

**Figure 2:**
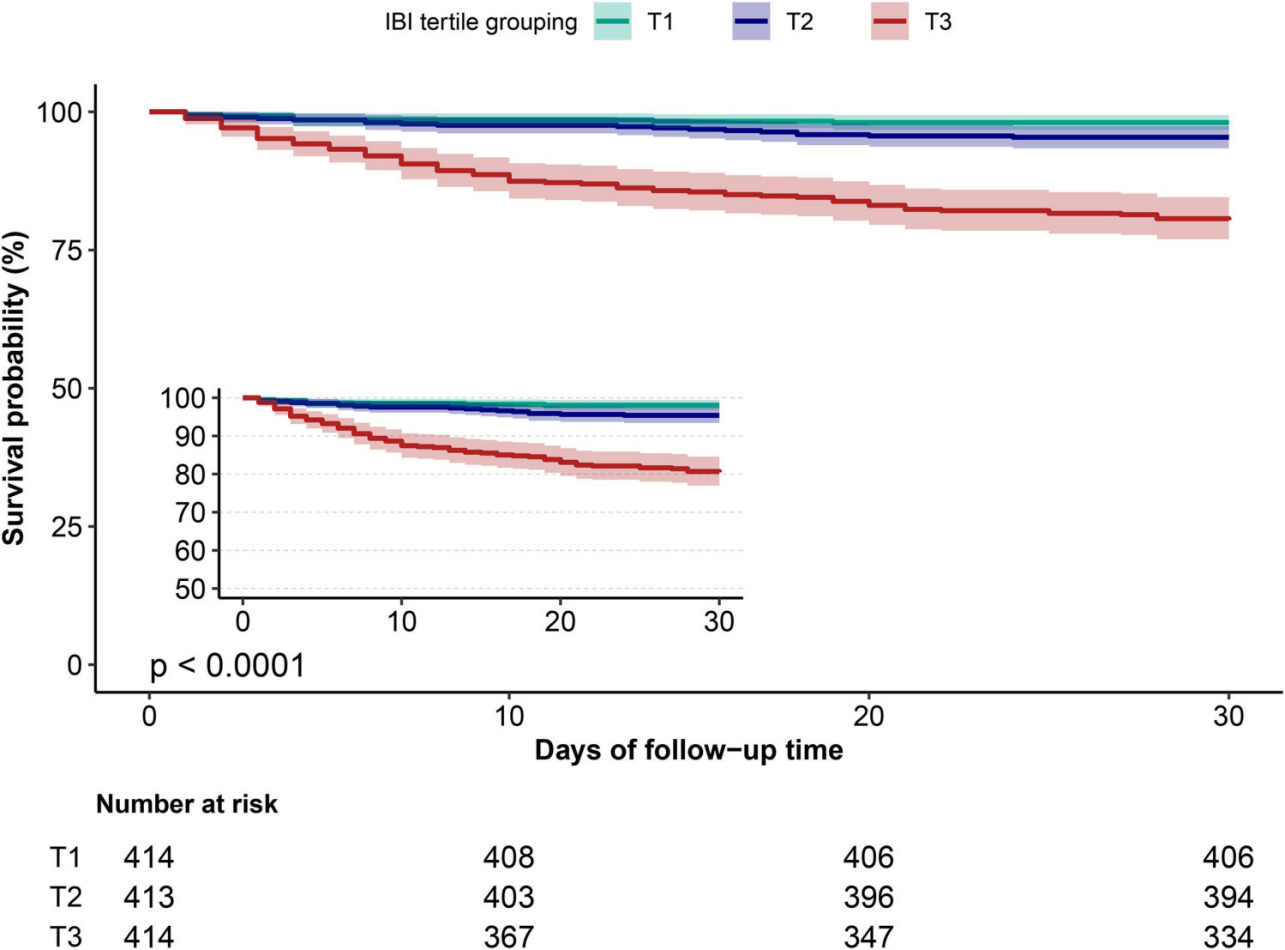
Cumulative survival rate curves of ADHF patients in IBI group. ADHF, acute decompensated heart failure; IBI, inflammatory burden index.

To assess the association between IBI and 30-day all-cause mortality in ADHF patients, we performed Kaplan–Meier analysis to plot survival curves for the three IBI groups. The significance of differences in survival rates among the groups was assessed using the log-rank test. Subsequently, we developed three adjusted Cox proportional hazards regression models to evaluate the association between IBI and 30-day mortality. Model 1 adjusted for baseline information assessed at admission, including sex, age, hypertension, diabetes, stroke, and CHD. Model 2 added NYHA classification, drinking status, smoking status, and LVEF. Based on Model 2, Model 3 was further adjusted for monocyte count, RBC, platelet count, AST, Cr, UA, TC, TG, HDL-C, LDL-C, FPG, and NT-proBNP. The proportional hazards assumption was evaluated using Kaplan–Meier curves for IBI groups and Schoenfeld residual tests, revealing no evidence of violation of this assumption (Figure 2 and Supplementary Figure S1). Additionally, based on collinearity assessments, we confirmed the absence of multicollinearity among covariates in the multivariable regression models (Supplementary Table S2) (35).

We also performed subgroup analyses to examine whether the association between IBI and 30-day mortality in ADHF patients was consistent across different subgroups. The subgroup variables and detailed stratification were as follows: age (<65 years vs. ≥65 years), sex (male vs. female), LVEF (<50% vs. ≥50%), NYHA classification (class III vs. class IV), hypertension (yes vs. no), diabetes (yes vs. no), stroke (yes vs. no), and CHD (yes vs. no). The significance of interaction effects was assessed using likelihood ratio tests.

To evaluate the predictive ability of IBI, we performed receiver operating characteristic curve analysis to assess the predictive performance of IBI and common inflammatory markers (CRP, neutrophil count, lymphocyte count, monocyte count, and WBC count) for 30-day mortality. The area under the curve (AUC), optimal threshold, sensitivity, and specificity were calculated for each indicator. Differences in AUCs were evaluated using the DeLong test. Additionally, we investigated the incremental predictive value of adding IBI to the established clinical risk model [Acute Decompensated Heart Failure National Registry (ADHFRE)] (36, 37) and calculated the C-index to quantify the improvement in predictive performance.

To ensure the robustness of the study findings, we conducted several sensitivity analyses: (i) Considering the potential impact of acute inflammation, we excluded patients with pulmonary infections at admission and repeated the primary analysis; (ii) To reduce the influence of reverse causality, we excluded participants who died within three days after admission; (iii) Given that hypertension, diabetes, stroke, and CHD are strong risk factors for adverse prognosis in ADHF patients, we excluded patients with these comorbidities and repeated the analysis (38, 39); (iv) For partially missing data (Supplementary Table S3), we performed multiple imputation to estimate missing values and repeated the primary analysis. (v) Medical treatment serves as the cornerstone of ADHF interventions. In subsequent models, we adjusted for ADHF treatment factors including beta-blockers, diuretics, ARB/ACEI/ARNI, and vasopressor agents. (vi) Considering that CHD patients undergoing PCI are typically a susceptible population for ADHF and do not interfere with the prognostic evaluation of IBI, we re-included this patient subgroup in further sensitivity analyses. (vii) To assess the generalizability of our findings, we utilized data from the United States National Health and Nutrition Examination Survey (1998–2018) to examine the association between IBI and all-cause mortality among participants diagnosed with congestive HF.

## Results

### Baseline characteristics

Among the 1,241 ADHF patients who met the study criteria, 720 were male and 522 were female, with a mean age of 68 years. The baseline characteristics of ADHF patients stratified by IBI tertiles are summarized in Table 1. Compared to patients in the low IBI group, those in the high IBI group were more likely to be male, older, and have a higher prevalence of diabetes, CHD, and NYHA Class IV. Additionally, they exhibited higher levels of CRP, WBC count, neutrophil count, monocyte count, AST, Cr, UA, FPG, and NT-proBNP, as well as lower levels of lymphocyte count, RBC count, TC, HDL-C, and LDL-C (All *p* < 0.05). Additionally, regarding treatment, compared to patients with low IBI, high IBI patients demonstrated a significantly lower proportion of ACEI/ARB/ARNI use (*p* = 0.015) and a markedly higher utilization rate of vasopressors (*p* < 0.001), while no significant differences were observed in diuretic or beta-blocker administration (both *p* > 0.05).

**Table 1:** Summary of baseline characteristics of the study population according to IBI tertiles group.

Variable	IBI tertiles			*P*-value
	Low (≤18.11)	Moderate (18.32–111.13)	High (≥111.30)	
No. of subjects	414	413	414	
Age (years)	68.00 (56.00–77.00)	70.00 (59.00–79.00)	74.00 (64.00–81.00)	<0.001
Sex (*n*, %)				<0.001
Male	203 (49.03%)	247 (59.81%)	269 (64.98%)	
Female	211 (50.97%)	166 (40.19%)	145 (35.02%)	
Hypertension (*n*, %)				0.347
No	242 (58.45%)	223 (54.00%)	241 (58.21%)	
Yes	172 (41.55%)	190 (46.00%)	173 (41.79%)	
Diabetes (*n*, %)				0.013
No	328 (79.23%)	299 (72.40%)	293 (70.77%)	
Yes	86 (20.77%)	114 (27.60%)	121 (29.23%)	
Stroke (*n*, %)				0.761
No	345 (83.33%)	339 (82.08%)	337 (81.40%)	
Yes	69 (16.67%)	74 (17.92%)	77 (18.60%)	
CHD (*n*, %)				0.020
No	309 (74.64%)	296 (71.67%)	273 (65.94%)	
Yes	105 (25.36%)	117 (28.33%)	141 (34.06%)	
NYHA classification (*n*, %)				<0.001
III	312 (75.36%)	268 (64.89%)	231 (55.80%)	
IV	102 (24.64%)	145 (35.11%)	183 (44.20%)	
Drinking status (*n*, %)				0.893
No	375 (90.58%)	378 (91.53%)	377 (91.06%)	
Yes	39 (9.42%)	35 (8.47%)	37 (8.94%)	
Smoking status (*n*, %)				0.034
No	360 (86.96%)	353 (85.47%)	334 (80.68%)	
Yes	54 (13.04%)	60 (14.53%)	80 (19.32%)	
Anti-heart failure treatment (*n*, %)				
Diuretic	401 (96.86%)	400 (96.85%)	401 (96.86%)	0.174
ACEI/ARB/ARNI	225 (54.35%)	242 (58.60%)	201 (48.55%)	0.015
Beta-blockers	311 (75.12%)	322 (77.97%)	299 (72.22%)	0.161
Vasopressor medications	102 (24.64%)	141 (34.14%)	221 (53.38%)	<0.001
LVEF (%)	51.00 (40.00–58.00)	47.00 (38.00–56.00)	50.00 (40.00–56.75)	0.015
CRP (mg/L)	2.58 (1.51–4.04)	9.09 (6.05–14.00)	59.65 (27.30–100.00)	<0.001
WBC (×10^9^/L)	5.50 (4.41–6.62)	6.60 (5.20–8.30)	8.00 (5.89–11.20)	<0.001
Neutrophil count (×10^9^/L)	3.50 (2.65–4.40)	4.74 (3.60–6.29)	6.32 (4.60–9.91)	<0.001
Lymphocyte count (×10^9^/L)	1.30 (1.00–1.70)	1.00 (0.77–1.40)	0.70 (0.49–1.04)	<0.001
Monocyte count (×10^9^/L)	0.41 (0.31–0.53)	0.50 (0.40–0.70)	0.59 (0.40–0.80)	<0.001
RBC (×10^12^/L)	4.14 (0.75)	4.06 (0.76)	3.81 (0.84)	<0.001
PLT (×10^9^/L)	162.00 (128.25–202.75)	167.00 (126.00–214.00)	164.00 (123.00–224.50)	0.618
ALT (U/L)	22.00 (14.00–34.50)	23.00 (14.00–42.00)	22.00 (14.00–43.00)	<0.001
AST (U/L)	25.00 (20.00–35.00)	26.00 (20.00–40.00)	30.00 (20.00–52.50)	<0.001
Cr (umol/L)	78.00 (63.00–99.00)	89.00 (71.00–124.50)	100.50 (76.00–155.50)	<0.001
UA (umol/L)	397.00 (313.50–480.00)	433.50 (336.00–558.25)	433.50 (327.75–562.75)	<0.001
TG (mmol/L)	1.14 (0.87–1.60)	1.13 (0.87–1.54)	1.12 (0.87–1.57)	0.940
TC (mmol/L)	3.80 (3.14–4.52)	3.80 (3.13–4.38)	3.58 (2.99–4.22)	0.005
HDL-C (mmol/L)	1.03 (0.84–1.20)	0.97 (0.80–1.19)	0.93 (0.72–1.14)	<0.001
LDL-C (mmol/L)	2.25 (1.78–2.90)	2.25 (1.81–2.88)	2.10 (1.69–2.62)	0.016
FPG (mmol/L)	5.20 (4.60–6.00)	5.30 (4.70–6.20)	5.80 (4.80–6.85)	<0.001
NT-proBNP (pmol/L)	2,838.00 (1,375.50–4,804.50)	3,723.00 (1,793.00–7,071.00)	4,256.00 (1,929.75–7,822.75)	<0.001
30-day mortality (*n*, %)	8 (1.93%)	19 (4.60%)	81 (19.57%)	<0.001

CHD, coronary heart disease; NYHA, New York heart association; LVEF, left ventricular ejection fraction; TG, triglyceride; TC, total cholesterol; HDL-C, high-density lipoprotein cholesterol; LDL-C, low-density lipid cholesterol; Cr, creatinine; WBC, white blood cell count; RBC, red blood cell count; PLT, platelet count; ALT, alanine aminotransferase; AST, aspartate aminotransferase; NT-proBNP, N-terminal pro-brain natriuretic peptide; UA, uric acid; FPG, fasting plasma glucose; CRP, C reactive protein; IBI, inflammatory burden index; ACEI, angiotensin-converting enzyme inhibitors ARB, angiotensin receptor inhibitors ARNI, angiotensin receptor neprilysin inhibitors.

### Follow-up

**Figure 3:**
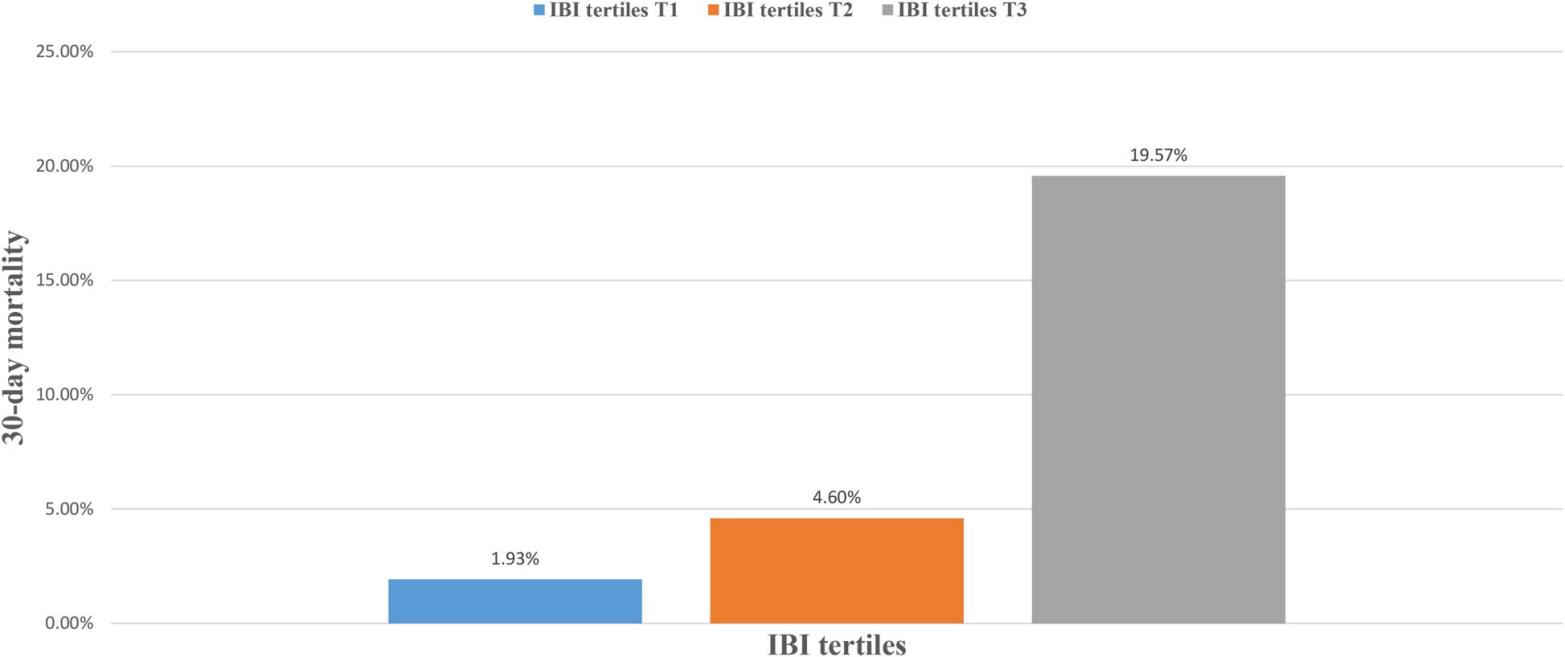
Bar chart showing 30-day mortality of ADHF patients stratified by IBI tertiles. ADHF, acute decompensated heart failure; IBI, inflammatory burden index.

During the 30-day follow-up, 108 deaths occurred among the 1,241 ADHF patients. The mortality rates in the low, moderate, and high IBI groups were 1.93%, 4.60%, and 19.57%, respectively (Figure 3): as IBI increased, the 30-day mortality rate among ADHF patients demonstrated a progressive increase. Kaplan–Meier analysis showed that higher IBI was associated with increased all-cause mortality: the high IBI group had a significantly higher 30-day mortality rate compared to the low and moderate IBI groups (Figure 2: log-rank *p* < 0.0001).

### Association between IBI and 30-day mortality in ADHF patients

Table 2 shows the hazard ratios (HRs) for the association between IBI, analyzed as both a continuous and categorical variable, and all-cause mortality. From Model 1 to Model 3, the HRs for the association between IBI and 30-day mortality in ADHF patients were 1.37, 1.40, and 1.29, respectively. Despite the attenuation of HRs with increasing levels of model adjustment, the positive association between IBI and 30-day mortality persisted across all models in ADHF patients. In the final model (Model 3), each SD increase in IBI was associated with a 29% increased risk of 30-day mortality (HR: 1.29, 95% CI: 1.15–1.46). Additionally, compared to the low IBI group, the high IBI group had a 368% higher risk of 30-day mortality (HR: 4.68, 95% CI: 1.06–13.73). Across all models, IBI showed a significant positive trend with 30-day mortality in ADHF patients (all *p*-trend < 0.001). These findings suggest that elevated IBI serves as an independent risk factor for poor short-term prognosis in ADHF patients.

**Table 2:** Multivariable cox regression analysis of the association between IBI and 30-day mortality in patients with ADHF.

Independent variable	Hazard ratios (95% confidence interval)			
	Unadjusted model	Model 1	Model 2	Model 3
IBI (Per SD increase)	1.40 (1.32, 1.48)	1.37 (1.29, 1.46)	1.40 (1.29, 1.52)	1.29 (1.15, 1.46)
IBI (tertiles)				
T1 (Low)	Ref	Ref	Ref	Ref
T2 (Moderate)	2.40 (1.05, 5.49)	2.16 (0.94, 4.95)	2.14 (0.89, 5.14)	2.13 (0.70, 6.53)
T3 (High)	11.02 (5.33, 22.79)	9.33 (4.48, 19.44)	8.15 (3.72, 17.88)	4.68 (1.60, 13.73)
*P*-trend	<0.0001	<0.0001	<0.0001	<0.0001

Model 1 adjusted for sex, age, hypertension, diabetes, stroke and CHD.

Model 2 adjusted for model 1 + NYHA classification, drinking status, smoking status, LVEF.

Model 3 adjust for: Model 2 + monocyte count, RBC, PLT, AST, Cr, UA, TC, TG, HDL-C, LDL-C, FPG and NT-proBNP.

ADHF, acute decompensated heart failure; IBI, inflammatory burden index; SD, standard deviation.

### Subgroup analysis

Table 3 presents the results of subgroup analyses stratified by age, sex, LVEF, NYHA classification, and comorbidities (hypertension, diabetes, stroke, and CHD). After further likelihood ratio tests, we found no significant interaction between IBI and 30-day mortality in ADHF patients across subgroups (LVEF, NYHA classification, and comorbidities; All *p*-interaction >0.05), except for sex. These findings indicate that the association between IBI and short-term mortality prognosis in ADHF patients demonstrates robust stability across the majority of patient populations. In the sex subgroup, females had a significantly higher risk of IBI-related all-cause mortality compared to males (HR: female1.52 vs. male1.33, *p*-interaction = 0.0431). By contrast, female patients with IBI-related ADHF demonstrated a 1.14-fold higher 30-day mortality risk compared to males.

**Table 3:** Stratified analysis showed the relationship between IBI and 30-day mortality in patients with ADHF in different age, sex, NYHA classification, LVEF and whether combined with hypertension/diabetes/stroke/CHD.

Subgroup	Adjusted hazard ratios (95% confidence interval)	*P* for interaction
Age (years)		0.4696
19–70	1.41 (0.97, 2.07)	
71–99	1.21 (1.06, 1.39)	
Sex		0.0431
Male	1.33 (1.17, 1.51)	
Female	1.52 (0.89, 2.59)	
NYHA classification		0.1900
III	1.63 (1.24, 2.14)	
IV	1.25 (1.10, 1.43)	
LVEF		0.4445
<50%	1.46 (1.03, 2.07)	
≥50%	1.26 (1.11, 1.44)	
Hypertension		0.1359
Yes	1.46 (1.36, 1.57)	
No	1.34 (1.23, 1.47)	
Diabetes		0.1058
Yes	1.18 (1.00, 1.40)	
No	1.39 (1.22, 1.59)	
Stroke		0.6535
Yes	1.28 (1.13, 1.46)	
No	1.35 (1.08, 1.67)	
CHD		0.9968
Yes	1.29 (0.84, 1.98)	
No	1.29 (1.14, 1.46)	

Abbreviations as in Table 1.

Models adjusted for the same covariates as in model 3 (Table 2), except for the stratification variable.

To further explore the potential clinical explanations for gender-based differences in IBI-associated mortality risk among ADHF patients, we performed a gender-stratified analysis comparing baseline comorbidities and treatment factors between medium-to-high IBI subgroups (IBI ≥18.32): Our findings revealed that compared to male ADHF patients, female ADHF patients exhibited a higher prevalence of diabetes and stroke but lower rates of hypertension and CHD. Regarding treatment, women were less likely to receive ACEI/ARB/ARNI and diuretics but more likely to receive beta-blockers and vasopressors compared to men. However, despite these observed trends, no statistically significant differences were found between genders in comorbidities or medication use (Supplementary Table S4, all *p* > 0.05).

### Predictive value of IBI and multiple common inflammatory markers for 30-day mortality

**Figure 4:**
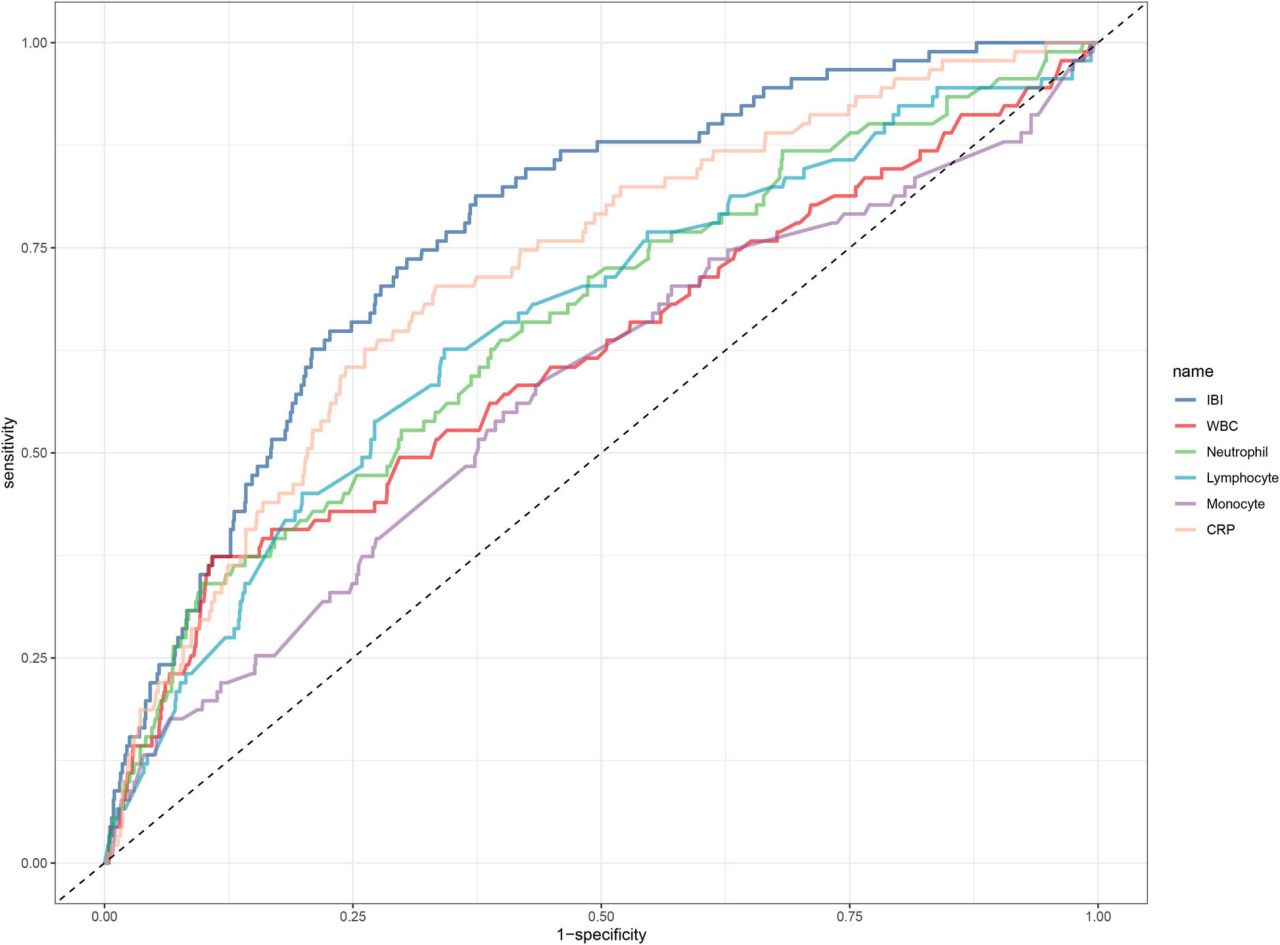
ROC analysis shows the predictive value of IBI and multiple common inflammatory markers on 30-day mortality in patients with ADHF. ROC, receiver operating characteristic curve; IBI, inflammatory burden index; WBC, white blood cell count; ADHF, acute decompensated heart failure.

The results of the predictive value analysis for IBI and multiple common inflammatory markers for 30-day mortality in ADHF patients are shown in Table 4 and Figure 4. The study demonstrated that conventional inflammatory biomarkers— CRP, WBC, neutrophil count, lymphocyte count, and monocyte count—each exhibited predictive value for 30-day mortality in ADHF patients, with respective predictive accuracies of 74%, 66%, 70%, 68%, and 60%. Compared with these conventional inflammatory biomarkers, IBI demonstrated superior predictive performance for 30-day mortality in ADHF patients, achieving approximately 80% accuracy (all DeLong's test *p* < 0.0001). Additionally, the optimal threshold for IBI in predicting 30-day mortality in ADHF patients was calculated as 159.86, with a specificity of 0.76 and a sensitivity of 0.70. Collectively, as a novel inflammatory biomarker, IBI significantly enhances the predictive accuracy for short-term adverse outcomes in ADHF patients beyond conventional inflammatory indicators.

**Table 4:** ROC analysis of IBI and various commonly used inflammatory indicators on the predictive value of 30-day mortality in ADHF patients.

Variable	AUC	95%CI low	95%CI upp	Best threshold	Specificity	Sensitivity
CRP*	0.74	0.70	0.79	23.65	0.74	0.66
WBC*	0.66	0.60	0.72	10.42	0.88	0.45
Neutrophil count*	0.70	0.64	0.75	8.75	0.89	0.43
Lymphocyte count*	0.68	0.63	0.74	0.84	0.65	0.67
Monocyte count*	0.60	0.54	0.66	0.55	0.60	0.56
IBI	0.80	0.75	0.84	159.96	0.76	0.70

* *P* < 0.001, compare with IBI.

AUC, area under the curve; ROC, receiver operating characteristic curve; WBC, white blood cell count; other abbreviations as in Table 1.

### Incremental predictive performance of IBI in mortality risk assessment

We further evaluated the incremental predictive value of adding IBI to the established clinical risk model (ADHFRE). The results demonstrated that incorporating IBI into the ADHFRE model for predicting 30-day mortality significantly improved its predictive performance: the C-index increased from 0.58 to 0.82 (*P* < 0.01). These findings highlight that the addition of IBI provides significant incremental value to the ADHFRE risk model for predicting short-term mortality.

### Sensitivity analyses

In the sensitivity analysis, the association between IBI and 30-day mortality in ADHF patients remained significant after excluding those with pulmonary infection (Table 5: Sensitivity-1); Specifically, in ADHF patients without pulmonary infection, IBI remained positively associated with 30-day mortality, yielding a HR of 1.61 (95% CI: 1.08–2.41). Furthermore, the main results remained largely unchanged after further excluding patients with hypertension, diabetes, stroke, and CHD, or patients who died within three days of admission: IBI maintained a robust positive association with 30-day mortality across these subgroups (Table 5: Sensitivity Analyses 2 and 3). Repeating the primary analysis in the imputed complete dataset yielded robust results (Table 5: Sensitivity-4). After adjusting for treatment factors including β-blockers, diuretics, ARBs/ACEIs/ARNI, and vasopressors, the findings remained consistent with the primary analysis (Table 5, Sensitivity-5). Additionally, we repeated the analyses in the complete cohort including patients who underwent PCI within the last 3 months (*n* = 1,241 + 44; due to missing CRP data in 58 participants who received PCI in the past three months, 44 additional individuals were incorporated into the original cohort, yielding a total of 1,285 participants). The findings remained consistent with the primary results (Table 5, Sensitivity-6). Finally, analysis of the external United States cohort confirmed a positive association between IBI and mortality risk among individuals with congestive HF (Table 5, Sensitivity-7), which is consistent with the results reported in the present study.

**Table 5:** Sensitivity analysis.

Independent variable	Hazard ratios (95% confidence interval)						
	Sensitivity-1	Sensitivity-2	Sensitivity-3	Sensitivity-4	Sensitivity-5	Sensitivity-6	Sensitivity-7
IBI (Per SD increase)	1.61 (1.08, 2.41)	1.36 (1.15, 1.60)	2.82 (1.76, 4.51)	1.25 (1.16, 1.35)	1.25 (1.08, 1.44)	1.28 (1.13, 1.44)	1.03 (1.01, 1.17)
IBI (tertiles)							
T1 (Low)	Ref	Ref	Ref	Ref	Ref	Ref	Ref
T2 (Moderate)	1.72 (0.30, 9.89)	1.43 (0.47, 4.34)	0.65 (0.12, 3.59)	1.54 (0.66, 3.58)	1.29 (0.49, 3.39)	1.09 (0.39, 3.01)	0.72 (0.36, 1.44)
T3 (High)	7.69 (1.57, 37.68)	4.09 (1.53, 10.93)	3.25 (0.65, 16.19)	4.62 (2.16, 9.86)	2.14 (0.85, 5.37)	3.64 (1.49, 8.87)	1.38 (0.72, 2.68)
*P*-trend	0.0074	0.0003	0.0669	<0.0001	0.0409	0.0001	0.0008

Sensitivity-1: Excluded patients with pulmonary infections at admission.

Sensitivity-2: Excluded participants who died within three days after admission.

Sensitivity-3: Excluded patients with combined hypertension, diabetes, stroke and coronary heart disease.

Sensitivity-4: Multiple imputation was used to handle missing data, and the association analysis was repeated.

Sensitivity-5: Further adjustments were made to key heart failure therapeutic agents including *β*-blockers, diuretics, ARBs/ACEIs/ARNI, and vasopressor medications.

Sensitivity-6: Repeated the analyses in the complete cohort including patients who underwent PCI within the last 3 months (*n* = 1,285).

Note 1: Adjusted for sex, age, hypertension, diabetes, stroke, CHD, NYHA classification, drinking status, smoking status, LVEF, monocyte count, RBC, PLT, AST, Cr, UA, TC, TG, HDL-C, LDL-C, FPG and NT-proBNP.

Note 2: Hypertension, diabetes, Cerebral stroke and CHD were not adjusted in Sensitivity-3.

Note 3: Sex, age, hypertension, diabetes, stroke, CHD, NYHA classification, drinking status, smoking status, LVEF, monocyte count, RBC, PLT, AST, Cr, UA, TC, TG, HDL-C, LDL-C, FPG NT-proBNP, *β*-blockers, diuretics, ARBs/ACEIs/ARNI, and vasopressor medications were adjusted in Sensitivity-5.

Note 4: Sex, age, hypertension, diabetes, stroke, CHD, drinking status, smoking status, monocyte count, RBC, PLT, AST, Cr, UA, TC, TG, HDL-C, LDL-C, FPG in Sensitivity-7.

## Discussion

This study is the first to investigate the association between IBI and 30-day mortality in a cohort of ADHF patients. The results demonstrate a significant positive association between IBI and 30-day mortality, with multiple sensitivity analyses further supporting the robustness of these findings.

Although the exact pathophysiological mechanisms of ADHF have not been fully elucidated, its deterioration is closely associated with significant activation of the neurohormonal system and inflammatory pathways (40–45). Notably, in patients with HF, elevated levels of inflammatory markers often precede increases in neurohormonal biomarkers (12, 46). This temporal pattern suggests that inflammatory-related indicators may provide earlier prognostic warning information for ADHF patients. IBI is a recently developed inflammatory index calculated by combining CRP, neutrophil count, and lymphocyte count. Numerous previous studies have demonstrated its potential utility in assessing the progression of various chronic diseases and cancer, highlighting its significant clinical applicability (17–33). For example, Du et al. demonstrated that in ischemic stroke patients undergoing endovascular thrombectomy, each SD increase in IBI was associated with a 74% higher risk of poor prognosis within 90 days (31). Findings from the National Health and Nutrition Examination Survey revealed a positive correlation between IBI levels and the prevalence of cardiovascular disease: compared to the low IBI group (Q1), the high IBI group (Q4) had a 43% increased risk of cardiovascular disease (33). Overall, high IBI is an important risk factor for inflammation-related diseases and their prognosis. Further validation is needed to determine whether these findings extend to other inflammation-related conditions. Moreover, the association between IBI and the prognosis of ADHF remains unclear. In this study, we examined the association between IBI and 30-day mortality in ADHF patients based on the Jiangxi-ADHF cohort. Our results demonstrate that IBI is an independent predictor of 30-day mortality prognosis in ADHF patients. Compared to those with low IBI levels, ADHF patients with high IBI levels exhibited a 368% higher risk of death within 30 days. This finding aligns with previously reported studies on IBI (17–33), demonstrating that elevated IBI levels exert adverse effects on health. In contrast, our study further expands the application of IBI and identifies it as a significant risk assessment factor for short-term mortality prognosis in ADHF patients. The predictive value of IBI in mortality risk has been extensively discussed in recent years. Existing studies have shown that IBI's predictive accuracy for survival rates in patients with various types of cancer ranges from 0.62 to 0.70 (8, 19, 23, 25, 27). In chronic inflammatory airway disease patients, IBI's predictive accuracy for all-cause mortality was 0.70, 0.67, 0.65, and 0.63 at 3, 5, 10, and 15 years, respectively (30). It is worth noting that in the assessment of non-mortality prognosis, Du et al. reported that IBI predicted 90-day adverse outcomes in acute ischemic stroke patients undergoing endovascular thrombectomy with an accuracy of 0.66 (31). In the current study, we analyzed the predictive performance of IBI for 30-day mortality in ADHF patients. The results showed that IBI had a predictive accuracy of 0.80, significantly outperforming conventional inflammatory markers such as CRP, WBC count, neutrophil count, lymphocyte count, and monocyte count. Similar findings have been reported by Song and Du et al., where IBI demonstrated the best predictive value for mortality outcomes in cancer and stroke patients compared to conventional inflammatory markers (18, 31). Based on IBI-related studies, we conclude that IBI is a superior novel inflammatory marker compared to conventional markers and demonstrates high predictive accuracy for short-term prognosis in acute diseases.

The mechanism by which high IBI leads to poor outcomes in ADHF patients remains unclear. However, based on the calculation method of IBI, it is evident that a high IBI implies elevated CRP, increased neutrophil count, and decreased lymphocyte count. Based on this background and literature review, we conducted the following analysis, which may provide insights into the mechanisms by which high IBI contributes to adverse outcomes in ADHF patients: (1) CRP is the most representative clinical marker of acute systemic inflammation. In HF patients, the interleukin-6–hsCRP pathway is significantly activated, leading to increased expression of inducible nitric oxide synthase and reduced cardiac contractility, ultimately resulting in poor short-term prognosis (47, 48). (2) Activated neutrophils release various proteolytic enzymes, including acid phosphatase, myeloperoxidase, and elastase. These enzymes can damage cardiomyocytes, exacerbating cardiac dysfunction and inflammatory responses, thereby worsening the prognosis of ADHF patients (49, 50). (3) In HF patients, visceral congestion can lead to intestinal lymphocyte loss, further impairing cardiac function and creating a vicious cycle of increasingly severe visceral congestion and decreased lymphocyte counts (51, 52). Based on the above analysis, we propose that a high IBI reflects a combined state of elevated CRP, increased neutrophil count, and decreased lymphocyte count. This comprehensive measure provides a more holistic reflection of the body's inflammatory and immune status, offering valuable prognostic information for clinical practice.

In the subgroup analysis, we observed a sex-specific association between IBI and ADHF prognosis: female ADHF patients exhibited a higher mortality risk than males at the same IBI level, suggesting that the inflammatory response may be more detrimental to female ADHF patients. This finding aligns with the “female survival disadvantage in HF” phenomenon reported in multiple studies. For instance, a Swiss cohort study including 5,825 HF patients demonstrated that females had a higher overall mortality risk regardless of the LVEF category (53). Furthermore, a multicenter study from Turkey also indicated that female acute HF patients had a significantly higher risk of in-hospital mortality compared to males (54). Regarding the sex-dependent association between IBI and ADHF prognosis, we propose that differences in sex-related pathophysiological mechanisms may be the core driving factors. Previous studies have shown that HF in males is often caused by macrovascular diseases (e.g., myocardial infarction) and myocardial structural remodeling, whereas females are more susceptible to coronary microvascular dysfunction, endothelial inflammation, and fibrosis (55, 56). These differences may amplify the detrimental effects of inflammation in female ADHF patients: On one hand, females often exhibit a stronger pro-inflammatory response during the acute phase of ADHF (e.g., more pronounced increases in CRP and interleukin-6), and the decline in estrogen levels (the mean age of the current study population was 68 years) may further diminish its anti-inflammatory protective effects (57–60). On the other hand, inflammatory mediators can synergistically exacerbate damage by interacting with female-specific pathological mechanisms, such as aggravating microvascular endothelial dysfunction and promoting myocardial fibrosis (55, 56). It should be noted that systemic inflammation interacts intricately with renal function, contributing to impaired iron metabolism and attenuated erythropoietin production/responsiveness, ultimately leading to anemia and iron deficiency (61). This anemia phenotype is more pronounced in female HF patients and correlates with significantly worse clinical outcomes (61–63). Additionally, psychosocial factors cannot be overlooked: the high prevalence of depression and anxiety may further exacerbate inflammatory cascades through neuroendocrine pathways (64, 65). These findings have dual implications for clinical practice: First, IBI may serve as a sensitive indicator for risk stratification in female ADHF patients, with high-IBI females prioritized for close monitoring and management. Second, treatment strategies for female patients should emphasize anti-inflammatory interventions.

One of the central challenges in cardiovascular medicine remains the high incidence of short-term adverse clinical outcomes among ADHF patients (9, 10). Addressing this clinical dilemma, the present study investigated the predictive performance of the novel inflammatory biomarker IBI for 30-day mortality risk in ADHF patients. Our findings demonstrate that IBI serves as an independent risk factor for short-term mortality in ADHF, exhibiting superior predictive value (AUC = 0.80) when compared with conventional inflammatory biomarkers. Notably, the simplicity of IBI measurement significantly enhances its clinical utility in emergency or inpatient settings, enabling timely identification of ADHF patients at high risk of adverse outcomes and facilitating early targeted therapies. We advocate for integrating automated IBI calculation algorithms within hospital electronic health record systems to optimize its clinical application. Consistent with previous IBI validation studies across diverse clinical contexts (17–33), these findings collectively underscore IBI's potential as a robust inflammatory biomarker with high generalizability.

### Strengths and limitations of the study

The strengths of this study lie in its novel findings and study population: (1) IBI showed excellent predictive value for 30-day mortality in ADHF patients (AUC: 0.80), which is promising news for ADHF patients as IBI can be obtained conveniently and effectively. (2) To our knowledge, this is the first study to evaluate the association between IBI and short-term mortality in ADHF patients, with results validated through multiple sensitivity analyses.

Some potential limitations should also be mentioned: (1) The participants in this study were primarily from Jiangxi, a southern city in China, which may restrict the applicability of our findings to northern China or other ethnic populations. (2) As a non-interventional study, it could not assess the impact of anti-inflammatory treatments on outcomes in ADHF patients after hospital admission. (3) This study primarily evaluated the predictive capability of IBI at admission for subsequent adverse events. The impact of IBI changes during hospitalization on prognosis is still unclear and warrants further investigation. (4) As with other observational studies, residual confounding cannot be eliminated. (5) A substantial proportion of ADHF patients lacked baseline CRP measurements at admission, resulting in missing IBI data. Although these missing values met the criteria for missing at random, the relative reduction in sample size may have influenced the findings to some extent, necessitating external validation in larger cohorts. (6) While 30-day follow-up effectively captures acute-phase events, it does not evaluate the longitudinal prognostic impact of IBI on ADHF patients. Future studies with extended follow-up are required to characterize the temporal trajectory of IBI's effects across short-, medium-, and long-term outcomes.

## Conclusion

This cohort study in Jiangxi, China, revealed a significant positive association between IBI and 30-day mortality in ADHF patients, emphasizing its predictive value. Incorporating IBI into the clinical management of ADHF patients may significantly help in preventing further disease progression.

## Data Availability

The raw data supporting the conclusions of this article will be made available by the authors, without undue reservation.
