# Supplementary material for: The impact of inflammatory burden index on the prognosis in acute decompensated heart failure: evidence from a cohort study in Jiangxi, China
Source: Front Cardiovasc Med. 2025 Oct 10;12:1604094. doi: 10.3389/fcvm.2025.1604094 (PMC12549653; doi:10.3389/fcvm.2025.1604094)
Supplement: Supplementary file 1 [file Datasheet1.pdf]

Supplemental Table 1: Comparing baseline characteristics of participants with and without missing IBI.

	IBI		*P*-value
	Missing	Non-Missing	
No. of subjects	1251	1551	
Sex			0.915
Male	726 (58.03%)	897 (57.83%)	
Female	525 (41.97%)	654 (42.17%)	
Hypertension (n,%)			0.356
No	713 (56.99%)	857 (55.25%)	
Yes	538 (43.01%)	694 (44.75%)	
Diabetes (n,%)			0.802
No	928 (74.18%)	1157 (74.60%)	
Yes	323 (25.82%)	394 (25.40%)	
Stroke (n,%)			0.131
No	1030 (82.33%)	1310 (84.46%)	
Yes	221 (17.67%)	241 (15.54%)	
CHD (n,%)			0.006
No	886 (70.82%)	1023 (65.96%)	
Yes	365 (29.18%)	528 (34.04%)	
NYHA classification (n,%)			0.227
III	819 (65.47%)	1049 (67.63%)	
IV	432 (34.53%)	502 (32.37%)	
Drinking status			0.689
No	1134 (90.65%)	1399 (90.20%)	
Yes	117 (9.35%)	152 (9.80%)	
Smoking status			0.996
No	1043 (83.37%)	1293 (83.37%)	
Yes	208 (16.63%)	258 (16.63%)	
Age (years)	71.00 (59.00-80.00)	71.00 (62.00-79.00)	0.265
LVEF (%)	49.00 (39.00-57.00)	45.00 (35.00-55.00)	<0.001
WBC ($\times 10^{12}$/L)	6.40 (5.06-8.51)	6.10 (4.80-7.80)	<0.001
Monocyte count($\times 10^{9}$/L)	0.50 (0.38-0.67)	0.50 (0.39-0.64)	0.676
RBC ($\times 10^{12}$/L)	4.00 (0.80)	4.09 (0.77)	0.004
PLT ($\times 10^{9}$/L)	164.00 (126.00-214.00)	164.00 (126.00-210.00)	0.172
ALT (U/L)	22.00 (14.00-40.00)	21.00 (14.00-36.00)	0.075
AST (U/L)	27.00 (20.00-40.50)	26.00 (19.00-38.00)	0.062
Cr (umol/L)	88.00 (69.00-126.00)	91.00 (71.00-126.00)	0.192
UA (umol/L)	419.00 (327.75-536.25)	438.00 (349.00-546.00)	0.004
TG (mmol/L)	1.14 (0.87-1.56)	1.14 (0.86-1.53)	0.634

TC (mmol/L)	3.71 (3.10-4.39)	3.78 (3.15-4.49)	0.258
HDL-C (mmol/L)	0.97 (0.78-1.17)	0.98 (0.80-1.18)	0.504
LDL-C (mmol/L)	2.21 (1.76-2.80)	2.23 (1.73-2.81)	0.739
FPG (mmol/L)	5.40 (4.70-6.40)	5.30 (4.70-6.20)	0.232
NT-proBNP (pmol/L)	3531.00 (1713.50-6409.00)	3743.00 (1997.00-6084.00)	0.234

Abbreviations: CHD: coronary heart disease; NYHA: New York Heart Association; LVEF: left ventricular ejection fraction; TG: triglyceride; TC: total cholesterol; HDL-C: high-density lipoprotein cholesterol; LDL-C: low-density lipid cholesterol; Cr: creatinine; WBC: white blood cell count; RBC: red blood cell count; PLT: platelet count; ALT: alanine aminotransferase; AST: aspartate aminotransferase; NT-proBNP: N-Terminal Pro-Brain Natriuretic Peptide; UA: uric acid; FPG: fasting plasma glucose; CRP: C reactive protein; IBI: Inflammatory Burden Index

Supplementary Table 2: Collinearity diagnostics steps.

	VIF		
	Step 1	Step 2	Step 3
IBI	2	2	2
Sex	1.3	1.3	1.3
Age	1.4	1.4	1.4
Hypertension	1.2	1.2	1.2
Diabetes	1.4	1.4	1.4
Stroke	1.1	1.1	1.1
CHD	1.1	1.1	1.1
NYHA classification	1.2	1.2	1.2
Drinking status	1.4	1.4	1.4
Smoking status	1.5	1.5	1.5
LVEF	1.3	1.3	1.3
CRP	1.6	1.6	1.6
WBC	339.5	NA	NA
Neutrophil count	322.4	2	2
Lymphocyte count	13.2	1.5	1.5
Monocyte count	3.1	1.4	1.4
RBC	1.5	1.5	1.5
PLT	1.3	1.3	1.3
ALT	6.3	6.3	NA
AST	6.3	6.3	1.1
Cr	1.5	1.5	1.5
UA	1.4	1.4	1.4
TG	1.7	1.7	1.7
TC	1.9	1.9	1.9
HDL-C	1.7	1.7	1.7
LDL-C	1	1	1
FPG	1.5	1.5	1.5
NT-proBNP	1.3	1.3	1.3

VIF: variance inflation factor; VIF = $1/(1-R^2)$. Abbreviations as in Table 1.

Note: The variables with VIF>5 will be regarded as collinear variables and cannot be included in the multiple regression model.

Supplementary Table 3. The missing number and rate of covariates.

	Non- Missing	Missing
Sex	1241	0
Age	1241	0
Hypertension	1241	0
Diabetes	1241	0
Stroke	1241	0
CHD	1241	0
NYHA classification	1241	0
Drinking status	1241	0
Smoking status	1241	0
LVEF	1195	46
CRP	1241	0
WBC	1241	0
Neutrophil count	1241	0
Lymphocyte count	1241	0
Monocyte count	1241	0
RBC	1241	0
PLT	1241	0
ALT	1225	16
AST	1225	16
Cr	1223	18
UA	1222	19
TG	1077	164
TC	1077	164
HDL-C	1077	164
LDL-C	1077	164
FPG	1192	49
NT-proBNP	1241	0
IBI	1241	0

Abbreviations as in Table 1.

Supplementary Table 4: Comparison of Baseline Comorbidities and Treatment Factors in Moderate-to-High IBI Population (IBI ≥18.32) Stratified by Sex.

	Male	Female	*P*-value
Hypertension			0.425
No	284 (55.04%)	180 (57.88%)	
Yes	232 (44.96%)	131 (42.12%)	
Diabetes			0.564
No	373 (72.29%)	219 (70.42%)	
Yes	143 (27.71%)	92 (29.58%)	
Stroke			0.433
No	426 (82.56%)	250 (80.39%)	
Yes	90 (17.44%)	61 (19.61%)	
CHD			0.120
No	345 (66.86%)	224 (72.03%)	
Yes	171 (33.14%)	87 (27.97%)	
Diuretic			0.171
No	18 (3.49%)	17 (5.47%)	
Yes	498 (96.51%)	294 (94.53%)	
ACEI/ARB/ARNI			0.709
No	237 (45.93%)	147 (47.27%)	
Yes	279 (54.07%)	164 (52.73%)	
Beta-blockers			0.565
No	132 (25.58%)	74 (23.79%)	
Yes	384 (74.42%)	237 (76.21%)	
Vasopressor medications			0.678
No	293 (56.78%)	172 (55.31%)	
Yes	223 (43.22%)	139 (44.69%)	

Abbreviations as in Table 1.
