# Supplementary material for: The impact of inflammatory burden index on the prognosis in acute decompensated heart failure: evidence from a cohort study in Jiangxi, China
Source: Front Cardiovasc Med. 2025 Oct 10;12:1604094. doi: 10.3389/fcvm.2025.1604094 (PMC12549653; doi:10.3389/fcvm.2025.1604094)
Supplement: Supplementary file 2 [file Image1.pdf]

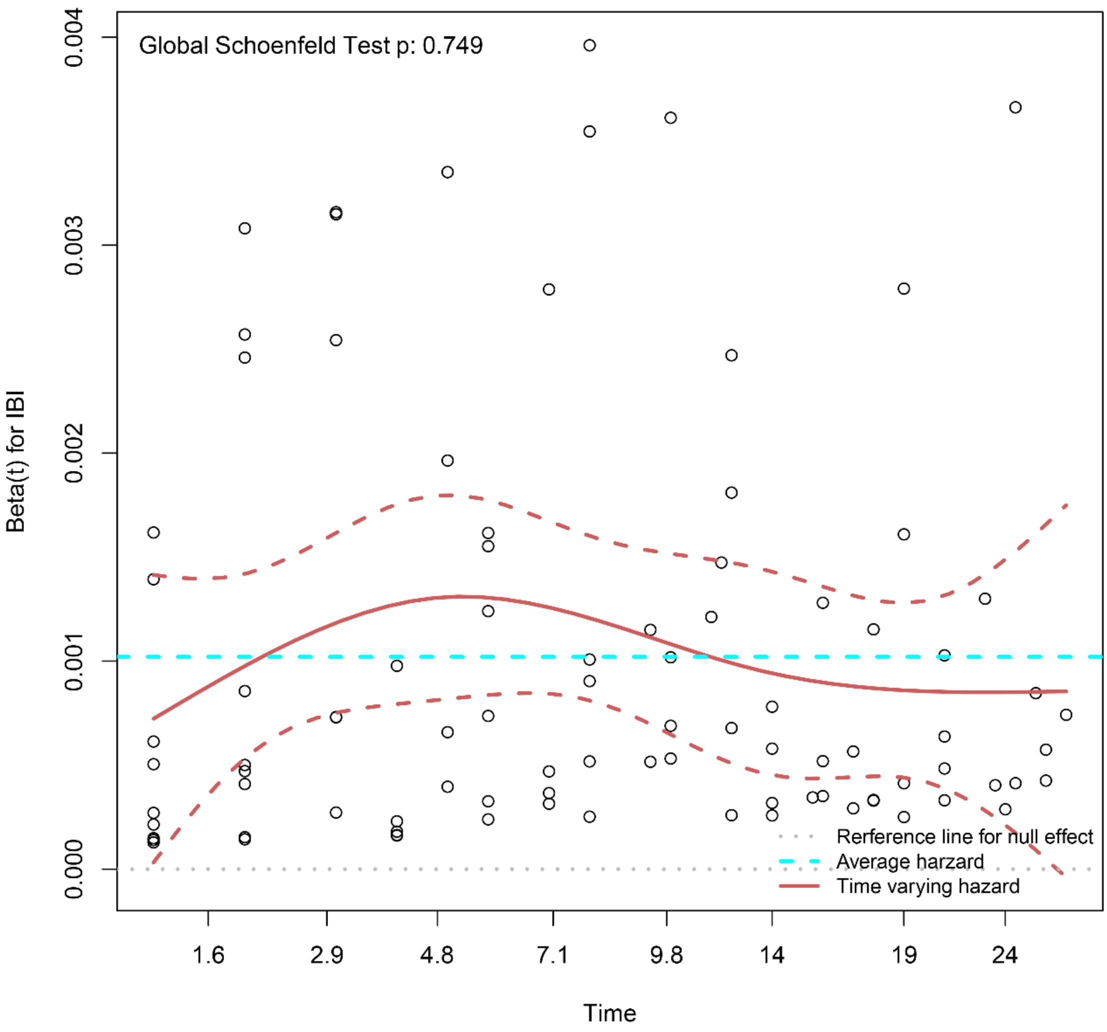

Supplementary Figure 1: Schoenfeld residual plot of IBI over time with 30-day mortality in ADHF patients as the dependent variable. The p-value of Schoenfeld Residuals Test result is larger than 0.05 which indicated that IBI is not a time dependent variable and can be analyzed by Cox Proportional Hazards Model.
